# Method of Calculating Short-Wavelength-Ratio-Based Color Temperature Supporting the Measurement of Real-Time Natural Light Characteristics through RGB Sensor

**DOI:** 10.3390/s20226603

**Published:** 2020-11-18

**Authors:** Seung-Taek Oh, Geon-Woo Jeon, Jae-Hyun Lim

**Affiliations:** 1 Smart Natural Space Research Center, Kongju National University, Cheonan 31080, Korea; ost73@kongju.ac.kr; 2 Department of Computer Science & Engineering, Kongju National University, Cheonan 31080, Korea; momentum96@smail.kongju.ac.kr; 3 Department of Urban Systems Engineering, Kongju National University, Cheonan 31080, Korea

**Keywords:** real-time natural light characteristics, short-wavelength ratio, color temperature, RGB sensor, measurement

## Abstract

The characteristics of natural light are mostly collected through specialized measuring equipment, such as a spectroradiometer, and some suggested measurement methods through a small RGB sensor. However, specialized measuring equipment presents difficulty in its high cost, and the RGB-sensor-based method has the limitation of being unable to measure the wavelength characteristics of natural light that are needed to implement lighting that supports circadian rhythms. This paper presents a method for calculating the short-wavelength-ratio-based color temperature of natural light in real time. First, an analysis of the correlation between the characteristics of natural light collected through a spectroradiometer was performed to determine the factors that were needed to accurately measure the color temperature of natural light. Then, the short-wavelength ratio of natural light was calculated through chromaticity coordinates (x and y), which are output values of the RGB sensor, and an equation for calculating the color temperature of natural light was derived through the short-wavelength ratio. Furthermore, after producing an RGB-sensor-based device, the derived equation was applied to calculate the color temperature of real-time natural light that reflects the wavelength characteristics. Then, as a result of the performance evaluation of the proposed method, the color temperature of natural light was accurately calculated within 1% of the average error rate.

## 1. Introduction

In the case of realizing lighting technology for better health, it is highly important to recognize the characteristics of natural light accurately [1,2]. Modern lighting technology is being developed to emphasize the functions of lighting, going beyond the simple preceding purpose of providing light. After LED lighting technology that could control the spectral characteristics was introduced, many studies were initially conducted on methods of controlling the illuminance of lighting, which considered energy efficiency [3,4]. However, studies are actively being conducted on controlling the color temperature and wavelength of lighting to implement a healthy light environment [5,6], and efforts to reflect the characteristics of natural light that are recognized as the most familiar and beneficial to humans are being continued [5,7]. A precedent study showed that the characteristics of a spectrum or the color temperature of natural light contributes to maintaining human body circadian rhythms [8,9] and showed that the characteristics of a certain short-wavelength band (460 or 446–488 nm) are related to sleeping and wake-up cycles [10,11,12]. Kim et al. analyzed the characteristics of natural light collected through an experimental test and suggested a standard for controlling the circadian color temperature of natural light to support the circadian rhythm [13]. Moreover, in the lighting field, LED light sources and lighting products for realizing the spectral characteristics of natural light have been developed [14]. Recently, a lighting product that provides different color temperatures according to the time of day, just like natural light, was released [15]. Additionally, the necessity of linking the control of indoor lighting with sensor technology that reacts to changes in the brightness and color temperature of outdoor light is being raised [16,17]. As such, studies are actively being conducted on lighting technology for realizing the characteristics of natural light, and the importance of accurate measuring and analyzing technologies for natural light is being emphasized [18]. Particularly, in the field of healthy lighting that supports circadian rhythms, the color temperature and short-wavelength property information of natural light, which change every moment, are very important [13]. In general, natural light is measured using specialized optical measuring equipment such as a spectroradiometer, through which the characteristics of the SPD (spectral power distribution), chromaticity coordinates, and color temperature can be gathered [19]. However, since there are difficulties in operating specialized optical measuring equipment due to the high costs and the necessity of expert knowledge for usage, general users cannot use it, and it cannot be applied to general lighting environments, which requires the application of sensors. In a precedent study, a device that applied a small image sensor was used to measure the SPD and calculate the correlated color temperature (CCT) [18]. Additionally, it presented a method of calculating the color temperature using the RGB pixel values of light source images taken by an HDR (high dynamic range) camera [20]. However, these methods also have limitations in that a relatively expensive image sensor and HDR camera need to be used. Although a method was recently introduced to easily measure color temperature by applying an RGB sensor, it has limitations in that it requires calibration to improve the accuracy of the measurement, and it only provides a limited range of spectral characteristics such as the chromaticity coordinates and color temperature [21,22,23]. For these reasons, an RGB-sensor-based measuring method is difficult to apply to the lighting technology field, which demands the measurement of wavelength characteristics to support circadian rhythms.

This paper proposes a method for calculating short-wavelength, ratio-based natural light color temperature that supports using an RGB sensor to measure the characteristics of natural light in real time. First, an analysis was performed on natural light characteristic DB (Database) gathered through an experimental test to determine the spectral characteristic factors needed to calculate accurate color temperatures that reflect the wavelength characteristics of natural light. Additionally, to measure the characteristics of natural light in a general lighting environment, a spectral characteristic measuring device configured with an RGB sensor and a diffuser was produced. Then, the RGB-sensor-based spectral characteristic measuring device and standard equipment (CAS 140CT) collected the characteristics of natural light in the same location and at the same time, and the characteristics were analyzed to derive a method for accurately estimating color temperature based on the short-wavelength ratio of natural light. This study presents a method for accurately calculating color temperatures based on the short-wavelength characteristics of natural light through an RGB sensor in a relatively easy way. Thus, it aims to contribute to implementing a healthy lighting system technology that reflects the characteristics of natural light in the future.

## 2. Characterization of Natural Light

In this study, a small RGB sensor was applied to measure and estimate the characteristics of real-time natural light, which is needed in the field of circadian rhythm-supporting lighting technology. For this, it was confirmed that wavelength characteristics can be derived based on spectral characteristics measured through the RGB sensor, and the correlation between the experimentally tested characteristics of natural light were analyzed to calibrate the measured results such as the color temperature. Natural light was measured on the rooftop of Kongju National University, which is located at a latitude of 36° and longitude of 127°, and a spectroradiometer (CAS 140CT, Instrument System, Munich, Germany) was applied as the measuring equipment. Table 1 indicates the main specifications of the spectrometer.

The experimental test for natural light was performed on all days from April 2017 to the present day, except on rainy days, and the spectral irradiance, illuminance, tristimulus values (X, Y, Z), chromaticity coordinates (x, y), and CCT (correlated color temperature) of natural light from sunrise to sunset were gathered. The spectral irradiance was measured in a wavelength band of 200–1000 nm, which is the instrumentation performance range of the spectroradiometer. Moreover, the color temperature of the light was determined from the characteristics of the wavelength, and the accurate color temperature of natural light can be confirmed through wavelength-based calculation. Thus, it was determined that factors that could replace spectral irradiance to indicate the spectral characteristics were necessary, and the band-by-band ratio of each short, middle, and long wavelength was calculated. At this point, each wavelength was classified into the following wavelength bands: 380–480 nm for short-wavelength, 480–560 nm for middle wavelength, and 560–780 nm for long wavelength. The band-by-band ratio of each wavelength was calculated using Equation (1) [13].

(1) $$\mathrm{SWR}\left(\mathrm{Short}\;\mathrm{Wavelength}\;\mathrm{Ratio}\right)=\int_{380}^{480}E_{\left(\lambda \right)}d\lambda ÷\int_{380}^{780}E_{\left(\lambda \right)}d\lambda \;\mathrm{MWR}\left(\mathrm{Middle}\;\mathrm{Wavelength}\;\mathrm{Ratio}\right)=\int_{480}^{560}E_{\left(\lambda \right)}d\lambda ÷\int_{380}^{780}E_{\left(\lambda \right)}d\lambda \;\mathrm{LWR}\left(\mathrm{Long}\;\mathrm{Wavelength}\;\mathrm{Ratio}\right)=\int_{560}^{780}E_{\left(\lambda \right)}d\lambda ÷\int_{380}^{780}E_{\left(\lambda \right)}d\lambda$$

**Figure 1:**
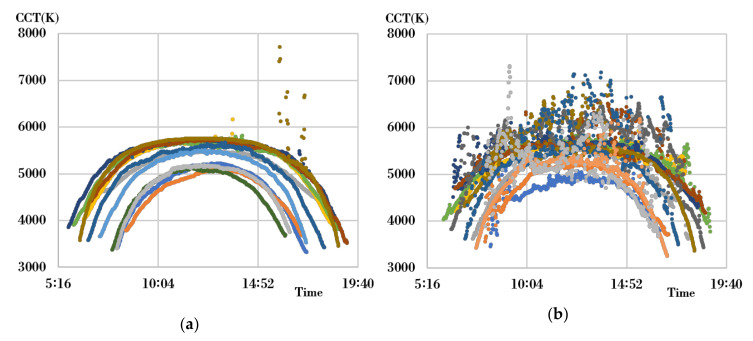
Hourly color temperature distribution of the experimentally tested natural light. (**a**) A clear day; (**b**) A cloudy day.

For the characterization of natural light, one clear day and one cloudy day were selected for each month among the data within the experimentally tested natural light characteristic DB to extract natural light characteristic data for a total of 24 days. At this point, clear days and cloudy days were separated using the measured results for color temperatures, which kept changing between sunrise and sunset. A clear day showed a relatively uniform arc-shaped change in color temperature per hour, while a cloudy day showed an irregular pattern. Considering this characteristic, the color temperature difference (hereafter referred to as the differentiated color temperature) was calculated each minute of the color temperature measurements on each day. Furthermore, in the entire color temperature section, the percentage of sections having a differentiated color temperature of more than 50 K was confirmed. A case where the ratio of sections having a differentiated color temperature of more than 50 K was within 2% was classified as a relatively clear day, and a case having the same of more than 2% was classified as a cloudy day. Figure 1 indicates the hourly color temperature distribution of the experimentally tested natural light during the total 24 days selected.

**Figure 2:**
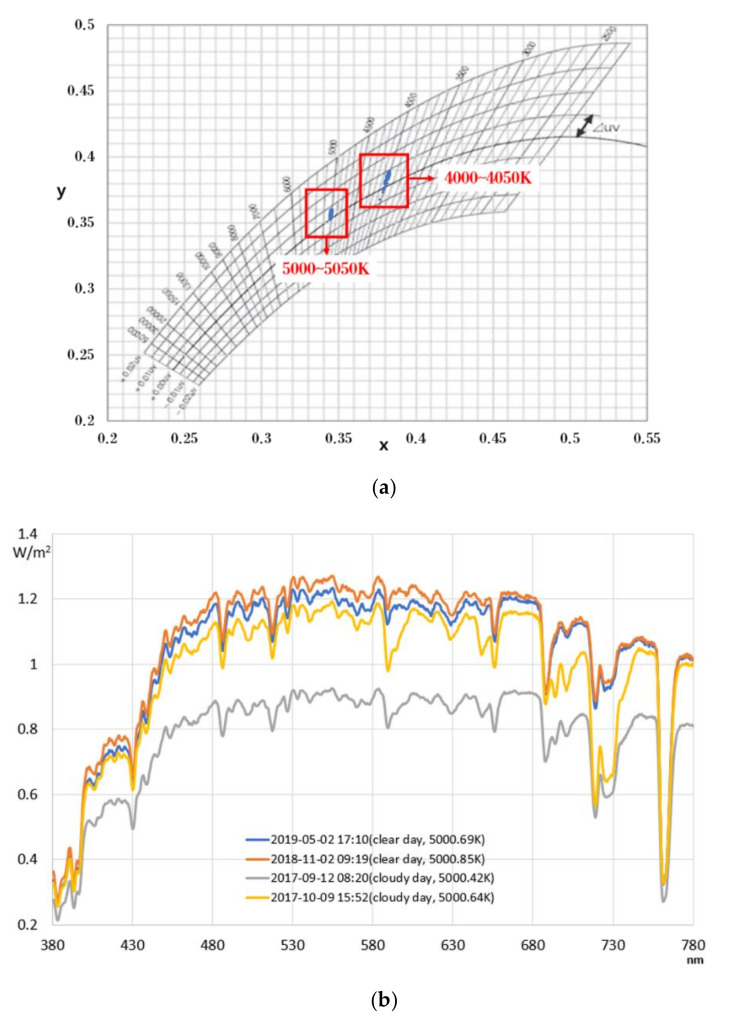
Characteristics of chromaticity and spectral power distribution in the same color temperature section. (**a**) xy chromaticity chart indicating the blackbody locus; (**b**) Spectral power distribution.

Figure 1a, which represents a clear day, shows an evenly distributed change in color temperature per hour in the range of 4000–6000 K, and Figure 1b, which represents a cloudy day, shows a rapidly changing and unstable pattern of change in color temperature in the range of 3800–8000 K. Figure 2 shows the characteristics of the chromaticity and wavelength distribution in certain color temperature sections for a better-detailed analysis of the spectral characteristics.

Figure 2a shows the chromaticity coordinates x and y of natural light at 4000–4050 and 5000–5050 K, which represent daytime, in color temperature sections of the general morning and night time along with spectral characteristics measured during the 24 days selected. It was confirmed that even in the same color temperature section, the chromaticity coordinates of natural light were not consistent, as they were widely distributed along the vertical direction. Figure 2b is the result of determining the SPD by extracting four instances (two instances for each clear and cloudy day) where the color temperature was 5000 K among the natural light characteristic data. It can be seen that although the color temperature of the natural light was the same, the characteristics of the spectrums can be different. Additionally, it was confirmed that when realizing natural light through artificial lighting in the future, the color temperature of the natural light determined from the wavelength characteristics shall be reproduced. Moreover, it was confirmed that although the spectral patterns were relatively uniform in the short-wavelength area (380–480 nm), they showed differences in SPD patterns in middle- and long-wavelength bands. To confirm the relationships among the characteristics of natural light in more detail, the correlation coefficients between each factor were analyzed. At this point, to also confirm how the characteristics of natural light were affected by the weather, clear and cloudy days were separated to analyze each correlation coefficient, and the results are shown in Table 2 and Table 3.

Table 2 shows the correlation coefficients among certain factors of natural light on clear days. For the color temperature of natural light, the short-wavelength ratio was 0.997, showing the highest correlation, and the ratio of the middle wavelength and chromaticity coordinates x and y was also more than 0.99, showing a high correlation. Furthermore, upon analyzing the correlation between the natural light’s short-wavelength ratio and chromaticity coordinates x and y, high negative correlations of −0.99 and −0.996 were found. According to the results on cloudy days shown in Table 3, the correlation between the natural light’s color temperature and short-wavelength ratio was 0.991, slightly lower than that on clear days. However, all the other spectral characteristics factors also showed a low correlation with color temperature on cloudy days, and in particular, the correlation between the tristimulus values X, Y and Z, illuminance and color temperature was quite low, with a value of 0.18–0.34. Additionally, the correlations between the short-wavelength ratio and chromaticity coordinates x, y and CCT were all above ±0.99, even on cloudy days, showing a similar correlation to that on clear days. Through the analysis of Table 2 and Table 3, it can be observed that while certain factors of natural light responded sensitively to changes in weather, the short-wavelength ratio, chromaticity coordinates x and y, and color temperature showed a relatively steady correlation. Through this, it was confirmed that a correlation formula linking the short-wavelength ratio, chromaticity coordinates and color temperature could be derived in the future.

## 3. A Method for Estimating Color Temperature of Real-Time Natural Light

In this study, to develop a method for accurately estimating the color temperature derived from the wavelength characteristics of natural light, an RGB-sensor-applying measuring device was first produced. Then, the characteristics of natural light were measured in the same environment by applying the standard equipment and the RGB-sensor-based device having different spectral characteristics factors that could be measured by each device. An analysis was conducted on natural light characteristics gathered through standard equipment and the RGB-sensor-based device, which have different performance, as well as measurable aspects of natural light. Through this, a method for estimating the color temperature of natural light based on the short-wavelength ratio was derived.

### 3.1. RGB-Sensing Device

**Figure 3:**
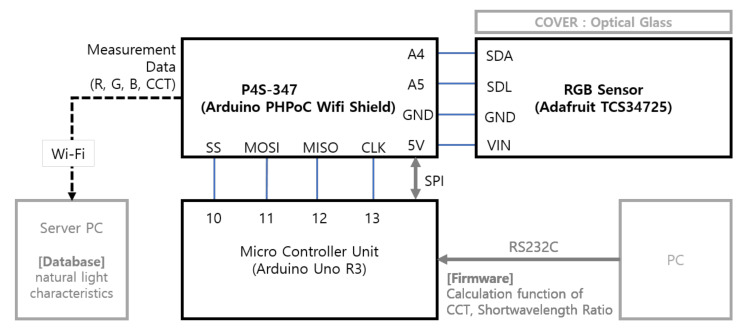
RGB-sensing device (block diagram).

An RGB-based device that could perform a real-time measurement of the color temperature of natural light, even in an outdoor environment, was produced. For this, an RGB sensor (TCS34725 Adafruit, Ams, Premstaetten, Austria) was selected as a sensor for measuring the color values of natural light first, and an Arduino Uno board was applied to implement the calculation, communication and control functions, which used the measured R, G and B values. Moreover, a PHPoC(PHP On Chip) shield was applied to establish a wireless communication (WiFi)-based transmission function for the results of the measurement and spectral characteristic estimation, and a diffuser was applied to prevent an inflow of excessive light intensity and to protect the sensor. Table 4 shows the main specifications of the RGB-sensing device, and Figure 3 shows the production outcome and a block diagram.

The RGB sensor in Table 4 measures the color properties of natural light within the illuminance range of 0–18,000 Lux and, then, outputs the result for each R, G and B value between 0 and 1000. However, the illuminance of natural light is more than 100,000 Lux on a clear day, and the saturation phenomenon in which the irradiance of the light exceeds the measurable range of the sensor could occur [18]. As measured, the results were not reliable when saturation occurred; a diffuser (optical glass) of a 10 mm standard was mounted on the upper area of the RGB-sensing part inside the produced device. This diffuser could measure the color temperature even when the illuminance was high, as it reduced the light penetration ratio. Additionally, the RGB-sensing device was configured to confirm the sensing results even through PCs or mobile devices, as it supports wireless communication (WiFi), and the MCU (Microcontroller Unit) was configured with the measured value-based spectral characteristic calculation function and transmission function of the estimated spectral characteristics. For the calculation function for spectral characteristics, the RGB color values received through the RGB sensor, based on Equation (2), were converted to the tristimulus values X, Y and Z, and Equation (3) was applied to calculate the chromaticity coordinates x and y. Furthermore, the McCamy formula of Equation (4) was applied to calculate the color temperature.

(2) Tristimulus values X= −0.14282×R+1.54924×G−0.95641×BTristimulus values Y= −0.32466×R+1.57837×G−0.73191×BTristimulus values Z= −0.68202×R+0.77073×G−0.56332×B

(3) $$\mathrm{Chromaticity}\;x=X\;/\;\left(X+Y+Z\right)\;\mathrm{Chromaticity}\;y=Y\;/\;\left(X+Y+Z\right)$$

(4) $$\mathrm{CCT}=444\times n^{3}+3525\times n^{2}-6823.3\times n+5520.33\;n=\left(x-0.3320\right)/\left(0.1858-y\right)$$

### 3.2. Short-Wavelength-Ratio-Based Color Temperature Calculation

The color temperatures of natural light derived from the wavelength characteristics can be calculated through calculation based on the spectral irradiance at different wavelengths gathered through the spectroradiometer [18]. However, in sensor technology s developed recently, a small RGB sensor supporting color temperature measurement was released, and methods for measuring spectral characteristics that apply this are being proposed. Nevertheless, the measurement of spectral characteristics, such as the color temperature, through an RGB sensor generally occurs through standard equipment and calibrations for a more accurate measurement result. At the time, methods for correcting the tristimulus X, Y and Z; the chromaticity coordinates x and y; and the color temperature itself were introduced. However, considering the high correlation among the short-wavelength ratio, chromaticity coordinates and color temperature of natural light, regardless of changes in weather during the analysis process for the natural light characteristics in Section 2, an equation for calculating these spectral characteristics was derived. For this, the characteristics of natural light were simultaneously and experimentally tested for a month through the previously produced RGB-sensor-based spectral characteristic measuring device and standard equipment (spectroradiometer, CAS 140CT). Table 5 shows the instruments and output spectral characteristics that can be obtained by each device.

**Figure 4:**
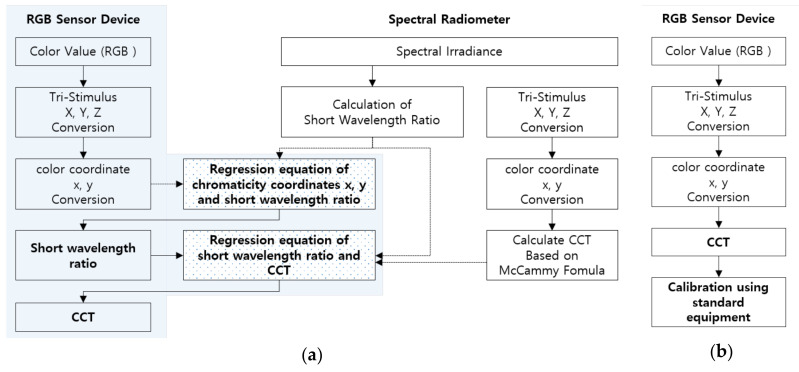
Color temperature calculation process: proposed method and existing method. (**a**) Proposed method; (**b**) Existing method.

After measuring the color temperature of natural light through the RGB-sensor-based color temperature-measuring device, a calibration process based on the measurements gathered through the standard equipment was performed. The most commonly used method at this point is performing calibration between the color temperatures of the two devices. Additionally, to further improve the measurement accuracy for the color temperature, methods for comparing and calibrating the result values between the tristimulus values or chromaticity coordinates are being applied [21,22,23]. However, the problem with these previous methods is that it is impossible to infer the wavelength characteristics of natural light, which is needed to implement healthy lighting, although they are able to measure the color temperature of natural light. This paper proposes a method for calibrating and calculating the color temperature of natural light using the short-wavelength ratio characteristics among the wavelength band-by-band (short, middle and long wavelengths) ratios, which were extracted based on the spectral irradiance gathered through the standard equipment. Figure 4 indicates the proposed method for calculating natural light’s short-wavelength-ratio-based color temperature. For comparison, the existing method for calculating the RGB-sensor-based color temperature is also shown.

By the existing method of Figure 4b, the tristimulus values, chromaticity coordinates and color temperature were calculated based on the measured color values, and calculated result values for the final color temperature were confirmed through calibration. However, in the proposed method of Figure 4a, a regression equation between the chromaticity coordinates x and y calculated through the RGB sensor and SWR (short-wavelength ratio) collected through the standard equipment was derived. Additionally, a regression equation between the short-wavelength ratio and color temperature collected through the standard equipment was derived. The equations for calculating the derived chromaticity-coordinate-based short-wavelength ratio and calculating the short-wavelength-ratio-based color temperature are Equations (5) and (6), respectively.

(5) $$\mathrm{SWR}\left(\%\right)=\;-256.321\times x+103.9395\times y+63.55018$$

(6) CCT= 149.66×SWR+2134

The method for calculating the short-wavelength-ratio-based color temperature was applied using Equations (5) and (6). This method was then applied to the previously produced RGB-sensor device. This replaced the existing RGB-sensor-based measuring method, which could simply have obtained color temperature measurements. Moreover, it provided color temperature measurements that reflected the short-wavelength ratio characteristics of natural light, which is essential for the circadian rhythm-supporting lighting technology recently emerging. Furthermore, this study aimed to present a color temperature calculation method that uses the short-wavelength ratio instead of using the existing calibration function, which uses the tristimulus values, chromaticity coordinates and color temperature.

## 4. Experiment and Discussion

**Figure 5:**
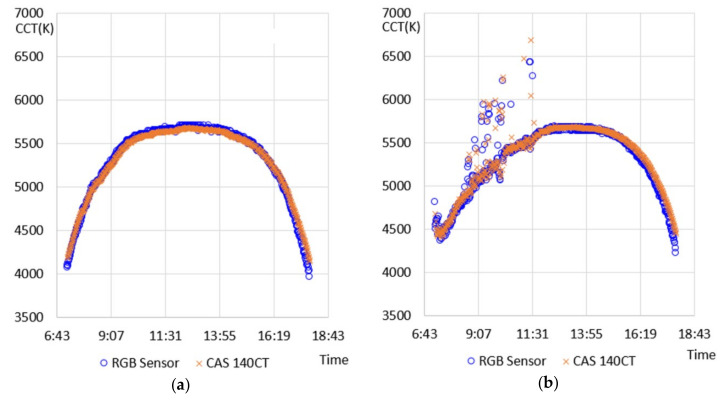
Comparison of measurement and calculation results for color temperatures. (**a**) Clear day (5 April 2020); (**b**) Cloudy day (4 April 2020).

An experiment was conducted for confirming the function of the proposed RGB-sensor-based spectral characteristic measuring device, which applied the method of calculating the short-wavelength-ratio-based color temperature. In the experiment, the RGB-sensing device and the standard equipment of the spectroradiometer (CAS 140CT) were used to measure the characteristics of natural light simultaneously in the same environment used for measuring natural light introduced in Section 2. A comparative experiment with the standard equipment was performed in April 2020, measurements were performed on three clear and three cloudy days (a total of 6 days, avoiding rainy days), and measurement and calculation results were collected. Figure 5 shows the measurement and calculation results for the color temperatures throughout the clear and cloudy days.

In the results on the clear day (Figure 5a), the standard equipment and the RGB-based device, which applied the proposed method, showed similar measurement result patterns. However, at 7 a.m. (sunrise +60 min) and at 6 p.m. (sunset −60 min), the color temperature measured through the proposed method showed a relatively lower value than the measurement result obtained with the standard equipment. Additionally, on the cloudy day (Figure 5b), a relatively larger difference between the results was indicated. However, it showed a similar measurement result in general, and between 7 and 11:30 a.m., when big changes in the color temperature were shown because of weather factors, such as clouds, the difference between the measurement results significantly increased. To further confirm the measurement function for the proposed method, measurement results obtained on all the comparative experimental days (8 days) were compared, and the results are shown in Table 6. Additionally, Table 6 compares the calculation results from applying the proposed method not only with the measurement results obtained through the standard equipment but also with the results obtained by calibration based on the color temperature and chromaticity coordinates, which was performed in the previous study.

According to the results displayed in Table 6, the average error rate for the color temperature measured through the RGB sensor was 34.06%, and when the color-temperature-based calibration was performed with the measurement results obtained with the standard equipment, the average error rate was 1.112%. In the case of applying the RGB sensor, it was confirmed that the calibration function is essential. In the case of performing chromaticity-coordinate-based calibration, the average error rate was 1.138%, indicating that it had no big differences from the color-temperature-based calibration method. However, in the case of applying the proposed calculation method for the short-wavelength-ratio-based color temperature, the average error rate was 0.903%, indicating that the most accurate calculation of the color temperature is possible with this method. Then, the standard deviations for the error rates were compared. As a result, it was confirmed that the proposed method calculated the color temperature in a relatively stable way compared to the other methods, as it showed the lowest value of 1.62%. The confirmed average error rate and the standard deviation of the error rate are the results that reflect the differences between the color temperatures in the section where the color temperature changes rapidly (Figure 5b). Hence, it can be determined that a more detailed calibration is required on the measured values in the relevant section, and it is considered that an additional performance improvement can be achieved through this. Furthermore, it was confirmed that the proposed method was also able to calculate the short-wavelength ratio (with an error rate of 5.31%), one of the spectral characteristics that could only have been confirmed through specialized equipment, such as the spectroradiometer, apart from by calculating the color temperature.

## 5. Conclusions

This paper proposes an accurate measurement method for color temperature, which is needed to establish healthy lighting technology. Particularly, to provide the color temperature that reflects the wavelength characteristics of natural light, required to control circadian rhythm-supporting lighting that reproduces natural light’s characteristics throughout a day, it presents a method for calculating the short-wavelength-ratio-based color temperature. For this, natural light DB collected through an experimental test was used to analyze the correlations among spectral characteristics, including natural light’s spectral irradiance, the ratios of different wavelengths (short, middle and long wavelengths), illuminance, tristimulus values (X, Y and Z), chromaticity coordinates (x and y) and color temperature. The analysis results indicate a high correlation of more than 0.99 among the chromaticity coordinates, short-wavelength ratio and color temperature. Then, a device that applied the RGB sensor (TCS34725) for the real-time measurement of the color values of natural light was produced. At this point, to prevent saturation that may occur when the illuminance of natural light is high at over 100,000 Lux, optical glass was placed on the upper part of the RGB sensor. Furthermore, the chromaticity coordinates x and y were entered to derive equations for accurately calculating the color temperatures. The equations include a regression equation linking the chromaticity coordinates x and y collected through the RGB sensor and the short-wavelength ratio of natural light collected through the spectroradiometer and a regression equation linking the short-wavelength ratio and color temperature, which were applied to the RGB-sensor-based measuring device. Additionally, to conduct a performance evaluation of the proposed method, a comparative experiment with the standard equipment of a spectroradiometer (CAS 140CT) was performed. Then, the error rates were compared with those found in the previous studies. As a result, error rates of 1.112% for the color temperature-based calibration method and 1.138% for the chromaticity-coordinate-based calibration method were shown. However, an error rate of 0.903% was shown for the proposed method, indicating that a calculation of color temperature more accurate than the previous methods can be achieved. Moreover, the short-wavelength ratio was also calculated in the color temperature calculation, and it showed an error rate of approximately 5% compared to that of the standard equipment. This study presents methods for applying a small RGB sensor to measure the color temperature and the characteristics of the short-wavelength ratio outside in real time for the first time. We aimed to develop a circadian rhythm-supporting lighting system that realizes not only the color temperature of natural light but also the short-wavelength ratio.

Continuous efforts will be invested in improving the measurement accuracy for color temperature, even in time zones with large changes in color temperatures such as before and after sunrise and sunset, or on cloudy days. Moreover, we will continue conducting studies to implement the proposed method by utilizing RGB sensors installed inside smartphones. Additionally, we are planning to conduct a study on developing a lighting system that supports maintaining the human body’s circadian rhythm, by applying the proposed method to determine real-time short-wavelength ratios and color temperature characteristics, through indoor artificial lighting.

## Figures and Tables

**Table 1:** Specifications of spectrometer (CAS 140CT).

Model (Company)	Image	Specifications
CAS 140CT-152 (Instrument Systems)	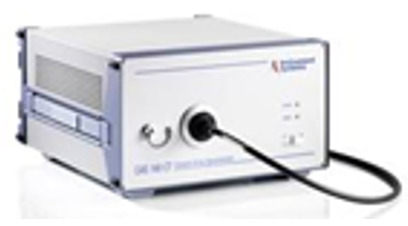	Spectral range (nm): 200–800 Number of pixels: 1024 × 128 Spectral resolution (nm): 2.7

**Table 2:** Results of analyzing correlation coefficients among certain factors of natural light: clear days.

Correlation Coefficient	SWR	MWR	LWR	tristimulus_X	tristimulus_Y	tristimulus_Z	color_coordinate_x	color_coordinate_y	Illuminance	CCT
SWR (Short Wavelengh Ratio)	1	−0.993	0.923	0.929	0.931	0.956	**−** **0.99**	**−** **0.996**	0.931	**0.997**
MWR (Middle Wavelengh Ratio)	−0.993	1	−0.961	−0.931	−0.933	−0.942	0.998	0.982	−0.933	−0.993
LWR (Long Wavelengh Ratio)	0.923	−0.961	1	0.885	0.886	0.86	−0.963	−0.896	0.886	0.928
tristimulus_X	0.929	−0.931	0.885	1	1	0.987	−0.921	−0.914	1	**0.92**
tristimulus_Y	0.931	−0.933	0.886	1	1	0.987	−0.923	−0.916	1	**0.922**
tristimulus_Z	0.956	−0.942	0.86	0.987	0.987	1	−0.931	−0.948	0.987	**0.948**
color_coordinate_x	−0.99	0.998	−0.963	−0.921	−0.923	−0.931	1	0.981	−0.923	**−** **0.991**
color_coordinate_y	−0.996	0.982	−0.896	−0.914	−0.916	−0.948	0.981	1	−0.916	**−** **0.992**
Illuminance	0.931	−0.933	0.886	1	1	0.987	−0.923	−0.916	1	0.922
CCT (color temperature)	0.997	−0.993	0.928	0.92	0.922	0.948	−0.991	−0.992	0.922	1

**Table 3:** Results of analyzing correlatn coefficients among certain factors of natural light: cloudy days.

Correlation Coefficient	SWR	MWR	LWR	tristimulus_X	tristimulus_Y	tristimulus_Z	color_coordinate_x	color_coordinate_y	illuminance	CCT
SWR (Short Wavelengh Ratio)	1	−0.994	0.922	0.279	0.289	0.433	**−** **0.991**	**−** **0.992**	0.289	**0.991**
MWR (Middle Wavelengh Ratio)	−0.994	1	−0.958	−0.305	−0.315	−0.449	0.994	0.979	−0.315	−0.984
LWR (Long Wavelengh Ratio)	0.922	−0.958	1	0.358	0.368	0.467	−0.946	−0.887	0.368	0.908
tristimulus_X	0.279	−0.305	0.358	1	1	0.981	−0.263	−0.24	1	**0.184**
tristimulus_Y	0.289	−0.315	0.368	1	1	0.983	−0.273	−0.25	1	**0.194**
tristimulus_Z	0.433	−0.449	0.467	0.981	0.983	1	−0.411	−0.401	0.983	**0.341**
color_coordinate_x	−0.991	0.994	−0.946	−0.263	−0.273	−0.411	1	0.987	−0.273	**−** **0.985**
color_coordinate_y	−0.992	0.979	−0.887	−0.24	−0.25	−0.401	0.987	1	−0.25	**−** **0.985**
Illuminance	0.289	−0.315	0.368	1	1	0.983	−0.273	−0.25	1	0.194
CCT (color temperature)	0.991	−0.984	0.908	0.184	0.194	0.341	−0.985	−0.985	0.194	1

**Table 4:** Main specifications of RGB-sensing device.

Part	Name	Specification
Color Sensor	Adafruit TCS34725 RGB Sensor	Sensing factor: red, green, blue, clear light sensing values; 3 × 4 photodiode array with IR-Blocking Filter; Measurable range: 0 to 65,535 for each RGB Channel Operating free-air temperature: −30–70 °C
MCU	Arduino Uno	Microcontroller: ATmega328P
WiFi Shield	P4S-348	PHPoC Shield for Arduino WLAN; topology: Ad-hoc, Infrastructure, Soft AP
Diffuser	Ground Glass	Diameter (mm): 10.00 ± 0.25; Thickness (mm): 1.60; Wavelength Range (nm): 350–2000

**Table 5:** Natural light characteristics that can be gathered by each device.

Classification	RGB-Sensor-Based Color Temperature Measuring Device	Standard Equipment (Spectroradiometer, CAS 140CT)
Instrument and output factors	Illuminance, RGB, tristimulus values (X, Y and Z), chromaticity coordinates (x and y), color temperature (CCT)	Spectral irradiance, wavelength band-by-band (short, middle and long wavelengths) ratios, illuminance, RGB, tristimulus values (X, Y and Z), chromaticity coordinates (x and y), color temperature (CCT)

**Table 6:** Comparison of color temperature measurement and calculation results.

		Color Temperature (K)					Short-Wavelength Ratio (%)	
Date	Time	(a) Standard EquipmentCAS 140CT	(Existing Method) RGB-Sensor-Based Measuring Device			(e) (Proposed Method) Short -Wavelength Ratio-Based Calculation	Standard EquipmentCAS 140CT	Proposed Method
			(b) Experimental Test Result (before Calibration)	(c) Color Temperature Calibration	(d) Chromaticity Coordinate-Based Calibration			
20.04.04	7:12	4681.87	6239.528	4265.26	3835.808	4807.789	16.64	16.85
20.04.04	7:13	4588.73	5815.352	4118.533	3773.164	4540.031	16.14	15.06
20.04.04	7:14	4518.28	5770.384	3953.319	3414.759	4501.389	15.76	14.80
20.04.04	7:15	4490.18	5910.468	3993.026	3649.885	4595.391	15.61	15.43
20.04.05	9:30	5388.59	7266.202	5376.623	5375.491	5402.430	21.70	20.82
20.04.05	9:31	5397.74	7328.484	5410.156	5408.558	5428.375	21.75	20.99
20.04.05	9:32	5410.07	7275.929	5381.86	5380.783	5406.809	21.82	20.85
20.04.05	9:33	5415.24	7275.929	5381.86	5380.783	5406.809	21.86	20.85
20.04.07	12:00	5459.03	7406.134	5451.963	5451.212	5463.187	22.52	21.23
20.04.07	12:01	5464.97	7442.926	5471.771	5472.471	5481.808	22.55	21.35
20.04.07	12:02	5470.72	7438.425	5469.348	5470.011	5479.863	22.58	21.34
20.04.07	12:03	5471.18	7429.341	5464.457	5465.046	5475.929	22.59	21.31
20.04.08	14:30	5628.06	7697.476	5608.821	5624.914	5619.363	23.26	22.27
20.04.08	14:31	5627.67	7798.847	5663.399	5681.245	5661.600	23.26	22.55
20.04.08	14:32	5626.42	7795.272	5661.474	5679.316	5660.274	23.25	22.54
20.04.08	14:33	5623.91	7726.041	5624.201	5641.772	5633.810	23.23	22.37
20.04.09	15:00	5427.2	7325.481	5408.539	5412.959	5440.796	22.09	21.08
20.04.09	15:01	5467.1	7386.821	5441.564	5448.513	5472.803	22.31	21.29
20.04.09	15:02	5506.61	7364.482	5429.537	5436.442	5463.224	22.50	21.23
20.04.09	15:03	5526.2	7423.004	5461.046	5468.895	5490.104	22.63	21.41
20.04.24	18:08	4171.02	5253.462	4292.964	4274.232	4151.222	13.12	12.47
20.04.24	18:09	4150.36	5345.677	4342.612	4321.42	4220.916	12.98	12.93
20.04.24	18:10	4122.84	5421.814	4383.605	4359.967	4276.069	12.82	13.30
20.04.24	18:11	4102.23	5345.801	4342.679	4319.52	4218.038	12.68	12.91
Average error rate (%)			34.06%	1.112%	1.138%	0.903%	-	5.31%
Standard deviation on error rate (%)			4.73%	1.78%	1.77%	1.62%	-	3.09%
